# Asymptotic Properties of Spearman’s Rank Correlation for Variables with Finite Support

**DOI:** 10.1371/journal.pone.0145595

**Published:** 2016-01-05

**Authors:** Petra Ornstein, Johan Lyhagen

**Affiliations:** Department of Statistics, Uppsala University, Uppsala, Sweden; National Institute of Environmental and Health Sciences, UNITED STATES

## Abstract

The asymptotic variance and distribution of Spearman’s rank correlation have previously been known only under independence. For variables with finite support, the population version of Spearman’s rank correlation has been derived. Using this result, we show convergence to a normal distribution irrespectively of dependence, and derive the asymptotic variance. A small simulation study indicates that the asymptotic properties are of practical importance.

## Introduction

A common question when looking at new data is “Does *Y* tend to increase when *X* increases?” When *X* and *Y* are ordinal, the nonparametric Spearman’s sample rank correlation, ${\hat{\rho }}_{s}$, is frequently used to measure the association.

Spearman originally thought of the situation where a small group of individuals are rated on two separate tasks [1]. His question was whether there existed an association between an individual’s two ratings. As *ρ*_*s*_ is defined as the sample correlation of the ranks of two variables this question translates to whether ${\hat{\rho }}_{s}$ is significantly different from zero. In cases when there are no ties, ${\hat{\rho }}_{s}$ follows a normal distribution under independence [2]. In practice, ${\hat{\rho }}_{s}$ is often used not for ratings, but for Likert type survey variables that take only a few values. When both variables are discrete with only a few categories, bias from not taking ties into account can become considerable with increasing sample size. In addition, the question of interest often concerns not only whether there exists an association but the size of that association. For example, the association between smoking and lung function has been heavily researched during the last half century. Both smoking and lung function are typically measured in categories, and the question of interest has over time shifted from whether smoking decreases lung function to the extent of the impact. In such cases, when ties cannot be disregarded or the research question is not posed against independence, an asymptotic distribution is lacking ([3], p. 7904).

The focus of this paper is on the properties of ${\hat{\rho }}_{s}$ when used as a measure of association between variables with finite support. [4] has constructed a population version of Spearman’s rho for discrete variables, *ρ*_*s*_. In this article, we apply Nešlehová’s results to the sample version of Spearman’s rank correlation, deriving its asymptotic properties and showing the importance of Nešlehová’s work to statistics.

In the next Section we introduce *ρ*_*s*_ and ${\hat{\rho }}_{s}$ for discrete variables with finite support. In Section three we derive the asymptotic properties of ${\hat{\rho }}_{s}$. Section four presents simulation results and some empirical examples. A conclusion ends the paper.

## Definitions

We are interested in the case when *X* and *Y* are discrete random variables with probability mass functions *p*_*i*_ = *P*(*X* = *i*) and *q*_*j*_ = *P*(*Y* = *j*) with finite support *i* ∈ {1, …, *I*}, and *j* ∈ {1, …, *J*}, *I*, *J* ∈ [2, ∞). Spearman’s sample rank correlation is typically seen in the following form

$${\hat{\rho }}_{s}=\frac{\sum_{i=1}^{n}\left(R_{i}-\bar{R}\right)\left(S_{i}-\bar{S}\right)}{\sqrt{\sum_{i=1}^{n}{\left(R_{i}-\bar{R}\right)}^{2}\cdot \sum_{i=1}^{n}{\left(S_{i}-\bar{S}\right)}^{2}}},$$ (1)

where *n* denotes the sample size and *R*_*i*_ = *rankX*_*i*_, *S*_*i*_ = *rankY*_*i*_, and $\bar{R}=\sum_{i=1}^{n}R_{i}/n=\left(n+1\right)/2=\bar{S}$.

Previous to Nešlehová’s work, Spearman’s sample correlation did not have a population version. In this Section we present Neslehova’s population version of Spearman’s rank correlation for variables that take a finite number of values [4]. In such cases, the relation between *X* and *Y* can be represented in a contingency table, and *ρ*_*s*_ can be written as a function of the cell probabilities. We denote the joint probability mass function *h*_*ij*_ = *P*(*X* = *i*∩*Y* = *j*). Then, $p_{i}=\sum_{j=1}^{J}h_{ij}$, and $q_{j}=\sum_{i=1}^{I}h_{ij}$. The cumulative marginal distribution functions are then $F_{i}=\sum_{k=1}^{i}p_{k}$ and $G_{j}=\sum_{k=1}^{j}q_{k}$ respectively. ([5], p. 94–95) defines Spearman rank correlation $\rho_{s}:R^{IJ-1}\to R$

$$\rho_{s}=\frac{3\sum_{i=1}^{I}\sum_{j=1}^{J}h_{ij}\;\left[\left(F_{i}+F_{i-1}\right)\left(G_{j}+G_{j-1}\right)-1\right]}{\sqrt{\left(1-\sum_{i=1}^{I}p_{i}^{3}\right)\left(1-\sum_{j=1}^{J}q_{j}^{3}\right)}}.$$ (2)

*ρ*_*s*_ is defined for cases with at least some variation in both *X* and *Y*, so that $\sum_{j=1}^{J}q_{j}^{3}<1$ and $\sum_{i=1}^{I}p_{i}^{3}<1$. We denote the empirical marginal distribution functions by $\hat{F}$ and $\hat{G}$, the estimated cell proportion in cell *i*, *j* by ${\hat{h}}_{ij}$ and let ${\hat{p}}_{i}=\sum_{j=1}^{J}{\hat{h}}_{ij}$, ${\hat{q}}_{j}=\sum_{i=1}^{I}{\hat{h}}_{ij}$. It turns out that the sample version of *ρ*_*s*_ equals the standard Spearman’s sample correlation. We thus have a second available expression of ${\hat{\rho }}_{s}$ ([4], p. 564)

$${\hat{\rho }}_{s}=\frac{3\sum_{i=1}^{I}\sum_{j=1}^{J}{\hat{h}}_{ij}\;\left[\left({\hat{F}}_{i}+{\hat{F}}_{i-1}\right)\left({\hat{G}}_{j}+{\hat{G}}_{j-1}\right)-1\right]}{\sqrt{\left(1-\sum_{i=1}^{I}{\hat{p}}_{i}^{3}\right)\left(1-\sum_{j=1}^{J}{\hat{q}}_{j}^{3}\right)}}.$$ (3)

## Asymptotic properties of ${\hat{\rho }}_{s}$

In this section we use the definitions presented above and apply the delta theorem to derive consistency, asymptotic unbiasedness, and asymptotic normality of ${\hat{\rho }}_{s}$ between variables with finite support.

As $\sum_{i=1}^{I}\sum_{j=1}^{J}h_{ij}=1$ there are only *IJ* − 1 unique probabilities and we can write $h_{IJ}=1-\sum_{i=1}^{I-1}\sum_{j=1}^{J-1}h_{ij}-\sum_{j=1}^{J-1}h_{Ij}$. Denote **h**_*IJ*_ = [*h*_11_, …, *h*_*IJ*_]^*T*^, and to avoid linear dependence, define the vector **h** = [*h*_11_, …, *h*_*I* − 1,*J*_]^*T*^ as the first *IJ* − 1 entries of **h**_*IJ*_.

**Theorem** If *X* and *Y* are discrete random variables with finite support, *ρ*_*s*_ is as defined in Eq (2), the gradient of *ρ*_*s*_ with respect to **h** is denoted by ${\dot{\rho }}_{s}$, and the covariance matrix of **h** is denoted by *Σ*, then

$$\sqrt{N}\;\left({\hat{\rho }}_{s}-\rho_{s}\right)\to N\;\left(0,{\dot{\rho }}_{s}^{T}\Sigma {\dot{\rho }}_{s}\right).$$ (4)

**Proof**. As shown by ([6], p. 419) $\sqrt{N}\left({\hat{h}}_{IJ}-h_{IJ}\right)$ converges in distribution to a singular multivariate normal distribution with mean zero, covariance matrix $diag\left(h_{IJ}\right)-h_{IJ}h_{IJ}^{T}$ and rank *IJ* − 1. It follows that $\hat{h}$ converges in probability to **h**. This implies that $\sqrt{N}\left(\hat{h}-h\right)$ converges in distribution to a nondegenerate multivariate normal distribution with mean zero, and covariance matrix *Σ* = *diag*(**h**) − **h** **h**^*T*^. As all terms in Eq (2) are functions of **h**, *ρ*_*s*_ can be consistently estimated from the cell proportions.

Next, we show that ${\hat{\rho }}_{s}$ is continuous with continuous first partial derivatives. Denote the separate terms of *ρ*_*s*_ as follows:

$$\begin{array}{c} A=\sum_{i=1}^{I}\sum_{j=1}^{J}h_{ij}\left(F_{i}+F_{i-1}\right)\left(G_{j}+G_{j-1}\right), \end{array}$$ (5)

$$\begin{array}{c} B=\sqrt{\left(1-\sum_{i=1}^{I}p_{i}^{3}\right)(1-\sum_{j=1}^{J}q_{j}^{3}}). \end{array}$$ (6)

Then

$$\rho_{s}=3\frac{A-1}{B}.$$ (7)

Since $\sum_{j=1}^{J}q_{j}^{3}<1$ and $\sum_{i=1}^{I}p_{i}^{3}<1$ we have that 0 < *B*^*k*^ < ∞, ∀*k*. *A* and *B* are simple functions of **h**, involving no division. Therefore, ${\hat{\rho }}_{s}$ is smooth with respect to $\hat{h}$, implying that application of the delta theorem to ${\hat{\rho }}_{s}$ is straightforward. We thus conclude that ${\hat{\rho }}_{s}$ converges to the distribution given in Eq (4).

For construction of the asymptotic covariance matrix, ${\dot{\rho }}_{s}$ is given below.

$${\dot{\rho }}_{s}=\frac{3\dot{A}-\rho_{s}\dot{B}}{B}.$$ (8)

where $\dot{A}=\frac{\partial A}{\partial h^{T}}$, $\dot{B}=\frac{\partial B}{\partial h^{T}}$, and for all (*r*, *s*) ≠ (*I*, *J*),

$$\begin{array}{ccc} \frac{\partial A}{\partial h_{rs}} & = & \left(F_{r}+F_{r-1}\right)\left(G_{s}+G_{s-1}\right)-\left(1+F_{I-1}\right)\left(1+G_{J-1}\right)\\ & & +\sum_{j=1}^{J}\left(G_{j}+G_{j-1}\right)\left[h_{rj}+h_{Ij}+2\sum_{i=r+1}^{I-1}h_{ij}\right]\\ & & +\sum_{i=1}^{I}\left(F_{i}+F_{i-1}\right)\left[h_{is}+h_{iJ}+2\sum_{j=s+1}^{J-1}h_{ij}\right], \end{array}$$ (9)

$$\frac{\partial B}{\partial h_{rs}}=\frac{3B}{2}\left[\frac{p_{I}^{2}-p_{r}^{2}}{1-\sum_{i=1}^{I}p_{i}^{3}}+\frac{q_{J}^{2}-q_{s}^{2}}{1-\sum_{j=1}^{J}q_{j}^{3}}\right]\text{.}$$ (10)

## A Monte Carlo experiment and empirical examples

In this section we first exemplify our results by a small Monte Carlo simulation and then by empirical examples. The Monte Carlo simulation is based on 20000 replicates for the sample sizes *n* = [50, 100, 200, 400, 800] and carried out in MATLAB version R2012b. For each replicate the data is generated as follows. First *n* observations are generated from a bivariate normal distribution with correlation 0.5. The variables are then discretized into five categories each such that the first variable has equal proportions, i.e. *p*_*i*_ = [0.2, 0.2, 0.2, 0.2, 0.2] and the second is skewed, *q*_*j*_ = [0.5, 0.25, 0.125, 0.0625, 0.0625]. This yields a population rank correlation *ρ*_*s*_ of 0.4249. In Table 1 the results from the Monte Carlo simulation are shown. In addition we run the simulation generating data from a bivariate normal distribution with correlation 0.95. The results from this simulation are consistent with those presented. In column one and two bias and mean square error of ${\hat{\rho }}_{s}$ are presented. From a practical perspective the bias is very close to zero. As the bias is close to zero the MSE is basically the variance, and as could be expected the MSE is halved when the sample size is doubled.

**Table 1:** Bias, MSE and rejection rates for the Spearman rank correlation. Rejection rates should be compared to the nominal 5%.

n	25	50	100	200	400	800
Bias	-0.0076	-0.0034	-0.0014	-0.0009	-0.0007	-0.0004
MSE	0.0301	0.0147	0.0072	0.0036	0.0018	0.0009
Rej-rate, Asymptotic	0.0931	0.0711	0.0609	0.0552	0.0513	0.0513
Rej-rate, Matlab	0.0405	0.0561	0.0903	0.1620	0.3252	0.6232
Rej-rate, Bootstrap	0.0602	0.0540	0.0527	0.0514	0.0496	0.0503

One way to analyze the normality of a statistic is to make a simple *z*—test at e.g. the 5% level. If the normality assumption is true then we would expect the rejection rate to be 5%. A 95-% confidence interval for a proportion of 0.05 is 0.047–0.053 for 20000 replicates. This means that observed proportions outside this interval would indicate that normality is not the case. In this part of the simulation we compare the asymptotic estimator with two other estimation strategies: the large sample approximation suggested by [7], available through e.g. MATLAB:s function corr and the empirical bootstrap. As the corr function does not give the variance but the *p*—value, the variance is solved from the formula of the *z*—statistic. The comparison with MATLAB:s built in function is chosen because it is easily available and therefore commonly used. However, this approximation disregards ties and is valid only under independence. We also analyze other approximations from the literature. They all rely on both the independence assumption as well as the assumption of continuous distribution, and they perform similarly to each other. Therefore, only the results from MATLAB:s built in function are shown. The bootstrap comparison is chosen because it tends to perform well and, although somewhat more complicated as well as computationally demanding to use, is typically a good choice in situations when a closed form for the variance is lacking.

From row three in Table 1 we see that the asymptotic variance is within the interval for sample sizes larger than 400 with good margin, indicating that normality, while an asymptotic property, is a good approximation for ${\hat{\rho }}_{s}$ from moderate sample sizes. The variance estimators used for comparison relate to the identical point estimate. From row four we see that violating the assumptions of independent and continuous observations has a severe impact on the results: MATLAB:s built in function performs poorly and does not improve with increasing sample size. The results from the bootstrap estimator (row five) are within the desired range by sample size 100, indicating that for small sample sizes, the bootstrap seems to be the best choice of variance estimator.

**Fig 1:**
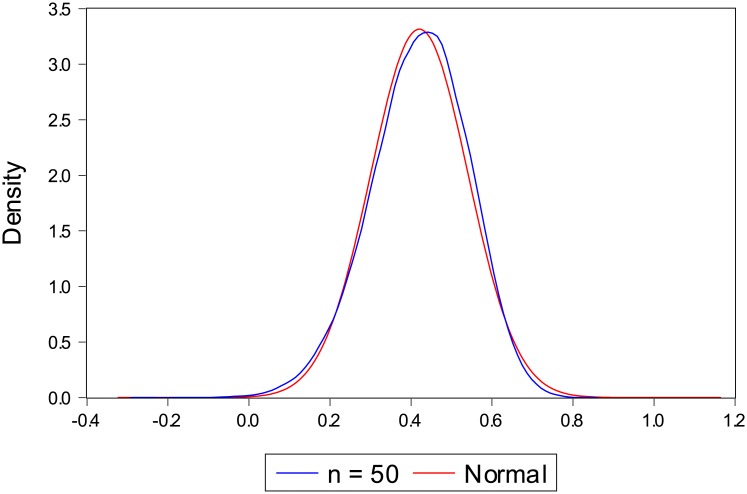
Kernel density of discrete version of Spearman rank correlation when sample size is 50 compared to a standard normal distribution.

A kernel density estimate of the small sample distribution for the sample size 50 is shown in Fig 1. A standard normal distribution is also shown as reference. The asymptotic variance seems to be fairly well approximated by the normal distribution although the empirical distribution has a slight negative skew. This deviation from normality is much lower for *n* = 100 and larger samples are very well approximated by the normal distribution. Due to space limitations, only *n* = 50 is displayed.

In the next step of the simulation study, we compare the power of the estimators. Variables are generated with the same characteristics as previously, but the correlation of the underlying continuous variables is now set to 0.55 and 0.65, yielding population rank correlations *ρ*_*s*_ of 0.4695 and 0.5608 respectively. The results are shown in Table 2. When the true rank correlation is 0.4695, no estimator exceeds a power of 0.36 even with a sample size of 800. When the true rank correlation is 0.5608, a larger difference from the null, the asymptotic estimator has a power of about 0.5 with a sample size of 100 and 0.95 with a sample size of 400. The asymptotic estimator consistently outperforms the bootstrap, but the difference is small and at least partly due to the bootstrap estimator’s somewhat lower rejection rates. Turning to MATLAB:s built in function, the results from Table 2 underscores those from Table 1 in showing that this type of estimator should not be used for other purposes than testing against *ρ*_*s*_ = 0.

**Table 2:** Rejection rates when testing against the null *H*_0_: *ρ* = 0.4249.

	Sample Size	25	50	100	200	400	800
*ρ*_*s*_ = 0.4695	Asymptotic	0.12	0.11	0.12	0.14	0.22	0.36
	Matlab	0.02	0.03	0.03	0.04	0.06	0.11
	Bootstrap	0.07	0.08	0.09	0.12	0.19	0.33
*ρ*_*s*_ = 0.5608	Asymptotic	0.26	0.33	0.49	0.73	0.95	1.00
	Matlab	0.02	0.03	0.05	0.09	0.21	0.46
	Bootstrap	0.16	0.25	0.41	0.68	0.93	1.00

We illustrate the performance of the three different types of estimators with empirical examples taken from [8] The results are shown in Table 3. The purpose is to give examples of the practical implications of the above derived asymptotic variance (*V*_*A*_), the bootstrap (*V*_*B*_), and MATLAB:s built in approximation (*V*_*M*_). *I* and *J* represent the number of values that *X* and *Y* can take respectively, and *n* gives the sample size. The sizes of the contingency tables and sample sizes are what is commonly encountered in empirical applications and the examples are from various fields: *2.4) income and job satisfaction, 2.11) inheritage of political views, 3.2) primary and secondary pneumonia infection in calves, 8.10) smoking and lung function*. The most striking result is that the asymptotic variance and the bootstrap estimates perform similarly, while *V*_*M*_ differs considerably. Returning to the correlation between smoking and decreased lung function (8.10), in the chosen example we have a point estimate of 0.24. Using our derived variance, we are 95 percent confident to say that this translates to a value in the interval (0.18; 0.30). The bootstrap estimate would similarly return a confidence interval of (0.18; 0.30), while the approximation assuming independence and no ties returns the wider interval (0.16; 0.32). One could think of a policy ascribing regulations to substances depending their established correlation with lung disease. For this, a hypothesis test with null hypothesis corresponding to the relevant threshold would be needed. In this case the use of a biased variance estimator would lead to an overestimation of uncertainty with a delay in health regulation as a potential consequence.

**Table 3:** Variance estimates and some other information for a few examples from [8].

Table	2.4	2.11	3.2	8.10 (40–59)
*ρ*_*s*_	0.102	0.523	0.402	0.240
*V*_*A*_	0.974	0.654	0.260	0.586
*V*_*M*_	0.998	0.875	0.932	0.975
*V*_*B*_	0.969	0.644	0.265	0.507
*I*	4	3	2	3
*J*	4	3	2	3
*n*	901	1852	156	654

## Conclusion

Using Nešlehová’s population version of Spearman’s rho we have been able to show that Spearman’s sample correlation has desirable asymptotic properties when applied to discrete variables. In particular, we have shown that ${\hat{\rho }}_{s}$ is consistent and asymptotically normal, and derived the asymptotic variance. Simulation results on both rejection rates and power indicate that the asymptotic variance performs as well as bootstrap for sample sizes from 400, allowing for easy construction of confidence intervals when Spearman’s correlation is used. For moderate to large sample sizes, the derived asymptotic variance combines the easy use of a closed form statistic with a performance on pair with the bootstrap. In addition, the existence of an asymptotic variance in closed form, suitable for practical applications, means that the potential uses of Spearman’s rank correlation in the construction of other estimators has increased.
